# Mono- and di-thiocarbamate inhibition studies of the δ-carbonic anhydrase TweCAδ from the marine diatom *Thalassiosira weissflogii*

**DOI:** 10.1080/14756366.2018.1450400

**Published:** 2018-03-26

**Authors:** Silvia Bua, Murat Bozdag, Sonia Del Prete, Fabrizio Carta, William A. Donald, Clemente Capasso, Claudiu T. Supuran

**Affiliations:** a Neurofarba Department, Sezione di Scienze Farmaceutiche e Nutraceutiche, Università degli Studi di Firenze, Florence, Italy;; b Department of Chemistry, Università degli Studi di Firenze, Florence, Italy;; c CNR, Istituto di Bioscienze e Biorisorse, Napoli, Italy;; d School of Chemistry, University of New South Wales, Sydney, Australia

**Keywords:** Carbonic anhydrase, metalloenzymes, mono-thiocarbamate, di-thiocarbamate, *Thalassiosira weissflogii*

## Abstract

The inhibition of the δ-class carbonic anhydrase (CAs, EC 4.2.1.1) from the diatom *Thalassiosira weissflogii*, TweCAδ, was investigated using a panel of 36 mono- and di-thiocarbamates chemotypes that have recently been shown to inhibit mammalian and pathogenic CAs belonging to the α- and β-classes. TweCAδ was not significantly inhibited by most of such compounds (K_I_ values above 20 µM). However, some aliphatic, heterocyclic, and aromatic mono and di-thiocarbamates inhibited TweCAδ in the low micromolar range. For some compounds incorporating the piperazine ring, TweCAδ was effectively inhibited (K_I_s from 129 to 791 nM). The most effective inhibitors identified in this study were 3,4-dimethoxyphenyl-ethyl-mono-thiocarbamate (K_I_ of 67.7 nM) and the *R*-enantiomer of the nipecotic acid di-thiocarbamate (K_I_ of 93.6 nM). Given that the activity and inhibition of this class of enzyme have received limited attention until now, this study provides new molecular probes and information for investigating the role of δ-CAs in the carbon fixation processes in diatoms, which are responsible for significant amounts of CO_2_ taken from the atmosphere by these marine organisms.

## Introduction

**Figure 1.**
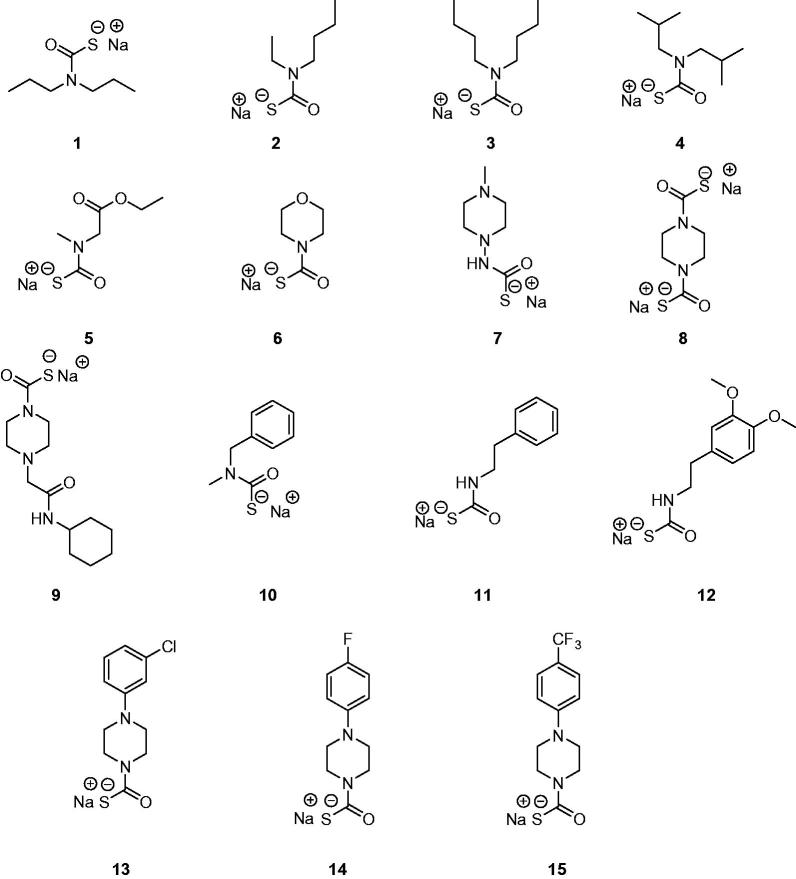
Monothiocarbamates (MTCs) **1**–**15** investigated as CA inhibitors^4^.

**Figure 2.**
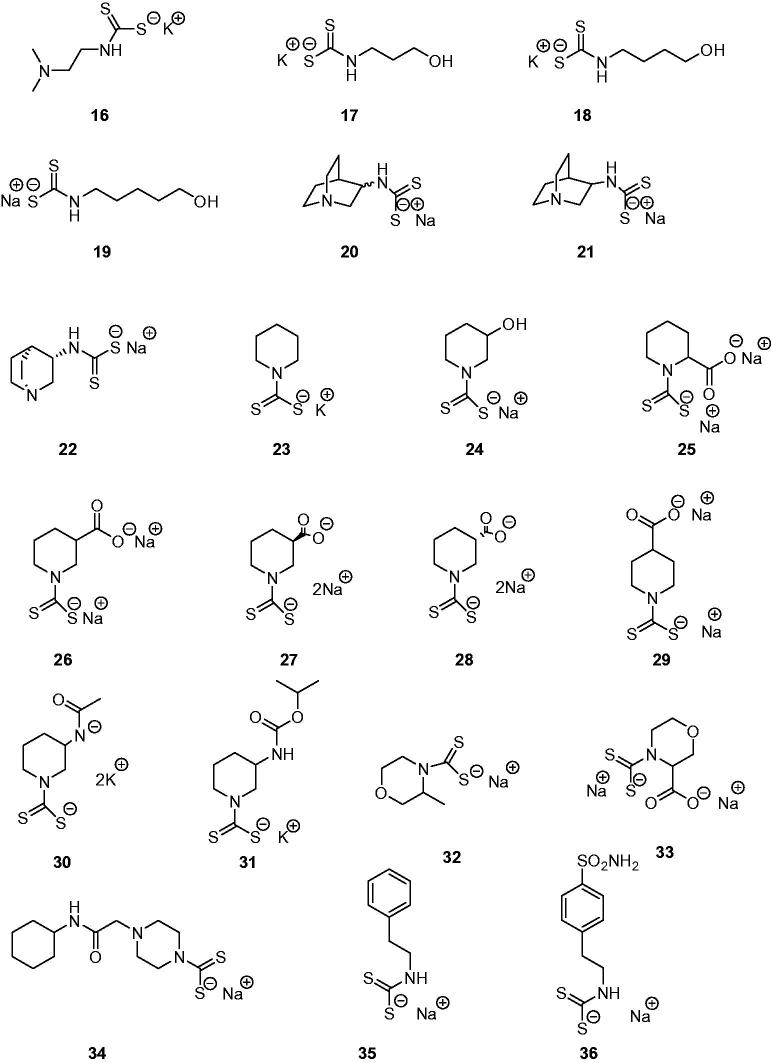
Dithiocarbamates (DTCs) **16**–**36** investigated as CA inhibitors^1^^,^^2^.

The di-thiocarbamates (DTCs) possessing the general formula RR^1^NCS_2_M (where R, R^1^ may be H, alkyl, cycloalkyl, aryl, hetaryl, etc., and M is a cation) were recently reported as a new class of inhibitors of the metalloenzyme carbonic anhydrase (CA, EC 4.2.1.1)^1^. Their inhibitory activity was investigated against α- and β-class CAs from various organisms^1^^,^^2^ and they also led to the discovery of two new CA inhibitor (CAI) classes, the xanthates^3^ and the mono-thiocarbamates (MTCs)^4^. Representatives of MTCs and DTCs acting as CAIs are shown in Figures 1 and 2.

Inhibition of CAs belonging to some of the seven genetically distinct families known to date^5–10^ has various biomedical applications owing to the fact that these enzymes catalyse a simple but physiologically crucial reaction: the hydration of CO_2_ to bicarbonate and hydronium ions^5–10^. Interference with this process has important physiological and pathological consequences because CAs are involved in pH regulation, biosynthetic processes, metabolism, secretion of electrolytes, transport of CO_2_/bicarbonate, etc.^5–10^. Their dysregulated expression or activity leads to various pathologies, and as a consequence, their inhibitors are clinically used as diuretics, antiglaucoma, antiepileptic, anti obesity, and antitumour agents^5–9^. Recently, the CAIs were also shown to be effective for the control of neuropathic pain, cerebral ischemia, and some forms of arthritis^10^. The primary sulphonamides and their isosteres (sulphamides and sulphamates) are the main class of CAIs, but in many cases, they indiscriminately inhibit most of the many CA isoforms known in an organism (e.g. 15 CA isoforms belonging to the α-class are known in humans^5^^,^^11–16^). This is the reason why alternative chemotypes, such as the DTCs and MTCs have recently been explored^1–4^. However, this class of CAIs has only been investigated to date for their interaction with human (h), α-class enzymes, and with several CAs from pathogens or model organisms, belonging to the α- and β-CA classes^1–4^. The δ-CAs were discovered in the diatom *Thalassiosira weissflogii*^6d^, but orthologues of this enzyme have been identified in most diatoms from natural phytoplankton assemblages and are responsible (along with other CAs) for CO_2_ fixation by marine organisms^17^. A related species of this diatom, *Thalassiosira pseudonana*, was shown to possess genes for three α-, five γ-, four δ-, and one ζ-CAs^18^. However, none of these enzymes have been cloned and characterised in detail to date, except TweCAδ^11^. Diatoms can be considered to be the organisms with the most intricate and poorly understood distribution of CAs, but the roles of these enzymes seem to be crucial for CO_2_ fixation and photosynthesis in many organisms and are estimated to be responsible for at least 25% of the inorganic carbon fixation in the oceans^6^^,^^17^^,^^18^. However, few studies are available for the interaction of δ-CAs with modulators of activity, inhibitors, and activators. TweCAδ was the only representative of the δ-class for which anion and sulphonamide inhibition studies have been reported to date^6d^^,^^11^. Here we report the first CA inhibition study with MTCs and DTCs of a δ-CA class enzyme, TweCAδ, which was cloned and characterised from the marine diatom *T. weissflogii*^6d^^,^^6e^.

## Materials and methods

### Materials

MTCs **1**–**15**^4^ and DTCs **16**–**36**^1^^,^^2^ were reported earlier by our group. Reagents/buffers of the highest available purity were obtained from Sigma-Aldrich, Milan, Italy. TweCAδ was a recombinant protein produced as reported earlier by our group^6e^^,^^11^.

### CA enzyme inhibition assay

An Sx.18Mv-R Applied Photophysics (Oxford, UK) stopped-flow instrument has been used to assay the catalytic activity of various CA isozymes for CO_2_ hydration reaction^12^. Phenol red (at a concentration of 0.2 mM) was used as indicator, working at the absorbance maximum of 557 nm, with 10 mM Hepes (pH 7.5) as buffer, and 0.1 M Na_2_SO_4_ (for maintaining constant ionic strength, which is not inhibitory against TweCAδ^11^), following the CA-catalysed CO_2_ hydration reaction for a period of 10 s at 25 °C. The CO_2_ concentrations ranged from 1.7 to 17 mM for the determination of the kinetic parameters and activation constants. For each inhibitor at least six traces of the initial 5–10% of the reaction have been used for determining the initial rate. The uncatalysed rates were determined in the same manner and subtracted from the total observed rates. Stock solutions of inhibitors (10 mM) were prepared in distilled-deionised diluted to 1 nM using the assay buffer. Inhibitor and enzyme solutions were pre-incubated together for 15 min (standard assay at room temperature) prior to assay, in order to allow for the formation of the enzyme inhibitor complex. The inhibition constant (K_I_), was obtained by considering the classical Michaelis–Menten equation and the Cheng-Prusoff algorithm by using non-linear least squares fitting as reported earlier^13–16^.

## Results and discussion

TweCAδ is the only CA belonging to the δ-class for which anion and sulphonamide inhibition studies were reported so far^6d^^,^^11^. Here, we investigated the inhibition of this enzyme with the panel of MTCs and DTCs of the types **1**–**36** shown in Figures 1 and 2. The results are shown in Table 1, where for comparison reasons, the inhibition of the human dominant isoforms hCA I and II with the same compounds are reported^1^^,^^2^^,^^4^.

**Table 1.** TweCAδ, hCA I, and hCA II Inhibition Data with MTCs **1**–**15**, DTCs **16**–**36**, and acetazolamide (**AAZ**, 5-acetamido-1,3,4-thiadiazole-2-sulphonamide) as standard drug, by a stopped-flow CO_2_ hydrase assay.

	RR^1^NCOS^−^ Na^+^ (**1**–**15**)		RR^1^NCS_2_M (**16**–**36**)		
			K_I_ (nM)^a^		
No.	R	R^1^	TweCAδ	hCA I	hCA II
**1**	*n*-Pr	*n*-Pr	806.7	>2000	46.7
**2**	Et	*n*-Bu	783.3	700	>2000
**3**	*n*-Bu	*n*-Bu	1142	909	>2000
**4**	*i*-Bu	*i*-Bu	>20,000	681	43.0
**5**	Me	CH_2_COOEt	>20,000	827	44.5
**6**		–(CH_2_CH_2_)–O–(CH_2_CH_2_)–	>20,000	569	>2000
**7**	H	–N(CH_2_CH_2_)N(CH_3_)CH_2_CH_2_–	487	>2000	35.0
**8**		– (CH_2_CH_2_)–NH-(CH_2_CH_2_)–	483	876	22.4
**9**		–(CH_2_CH_2_)-N(CH_2_CONHC_6_H_11_)–(CH_2_CH_2_)–	129	949	45.9
**10**	Me	CH_2_Ph	>20,000	>2000	>2000
**11**	H	CH_2_CH_2_Ph	997	>2000	43.7
**12**		HCH_2_CH_2_(3,4-di-MeO-C_6_H_4_)	67.7	891	26.7
**13**		– (CH_2_CH_2_)–N(3-Cl-C_6_H_4_)– (CH_2_CH_2_)–	1505	686	>2000
**14**		–(CH_2_CH_2_)–N(4-F-C_6_H_4_)–(CH_2_CH_2_)–	1498	895	46.8
**15**		–(CH_2_CH_2_)-N(4-CF_3_-C_6_H_4_)– (CH_2_CH_2_)–	1152	>2000	43.6
**16**	Me_2_N(CH_2_)_2_	H	8406	85.9	35.8
**17**	HO(CH_2_)_3_	H	8691	706	41.7
**18**	HO(CH_2_)_4_	H	7168	295	24.3
**19**	HO(CH_2_)_5_	H	8597	66.5	17.3
**20**		H	>20,000	494	48.7
**21**	(*R*)	H	>20,000	240	18.9
**22**	(*S*)	H	7995	615	65.9
**23**	–(CH_2_)_5_–	–	>20,000	252	30.1
**24**	–(CH_2_)_3_–CH(OH)CH_2_–	–	>20,000	428	60.7
**25**	–(CH_2_)_4_–CH(COONa)–	–	>20,000	485	80.1
**26**	–(CH_2_)_3_–CH(COONa)CH_2_–	–	8429	290	45.4
**27**	(*R*)–(CH_2_)_3_–CH(COONa)CH_2_–	–	93.6	496	80.5
**28**	(*S*) -(CH_2_)_3_–CH(COONa)CH_2_–	–	556	109	8.9
**29**	–(CH_2_)_2_–CH(COONa)(CH_2_)_2_–	–	8980	337	78.7
**30**	–(CH_2_)_3_-CH(NHAc)CH_2_–	–	783	910	47.9
**31**	–(CH_2_)_3_-CH(NHBoc)CH_2_–	–	9239	683	13.2
**32**	–CH(Me)CH_2_-O-(CH_2_)_2_–	–	>20,000	434	60.2
**33**	–CH(COONa)CH_2_-O– (CH_2_)_2_–	–	>20,000	84.7	78.5
**34**	–	–(CH_2_)_2 _N(CH_2_CONHC_6_H_11_)(CH_2_)_2_–	791	415	67.2
**35**	Ph(CH_2_)_2_	H	897	425	107
**36**	–	H_2_NO_2_SC_6_H_4_(CH_2_)_2_H	704	97.5	48.1
**AAZ**	–	–	83	250	12.1

a Mean ± standard error (from three different assays), by a stopped-flow technique (errors were in the range of ±5–10% of the reported values).

The following structure-activity relationship (SAR) can be obtained from the data of Table 1:

(i) A number of MTCs, including **4**–**6**, **10** and the DTCs **20**, **21**, **23**–**25**, **32**, and **33**, did not inhibit TweCAδ up to 20 µM, although many of these compounds were rather effective inhibitors of hCA I and/or hCA II (Table 1). Such MTCs/DTCs inhibitors are classified as aliphatic, heterocyclic, aromatic, or polycyclic types. Given the structural diversity of such compounds and high inhibition constants, it is challenging to delineate the SAR.

(ii) The MTCs/DTCs **3**, **13**–**19**, **22**, **26**, **29**, and **31** were relatively ineffective inhibitors of TweCAδ with inhibition constants in the micromolar range (K_I_s ranged between 1142 and 9239 nM; Table 1). These compounds are also highly heterogeneous. The main observation of these data is that the identity of the zinc-binding group, ZBG (MTC or DTC), does not significantly impact the activity of TweCAδ.

(iii) The MTC/DTCs **1**, **2**, **7**–**9**, **28**, **30**, and **34**–**36** were relatively effective inhibitors of TweCAδ, with inhibition constants in the range of 129–997 nM (Table 1). Some of the MTC and DTCs incorporate the piperazine ring (**7**–**9**, **34**). In addition, MTC **9** and DTC **34** have the same scaffold but a different ZBG. In this particular case, MTC **9** inhibited TweCAδ 6.1-times more efficiently than DTC **34**. Interestingly, for the β-CAs, the MTCs were usually much weaker inhibitors compared to the structurally similar DTCs^4^. In addition, the sulphonamide-containing DTC **36** (which contains two potential ZBGs, the sulphonamide and the DTC), there are no net differences of TweCAδ inhibitory activity compared to the structurally similar derivatives (e.g. **35**) which probably is due to the fact that the DTC in **36** is primarily binding to the metal ion in the enzyme active site, and not the sulphonamide moiety. However, the heterocyclic sulphonamide acetazolamide (**AAZ**, 5-acetamido-1,3,4-thiadiazole-2-sulphonamide), a clinically used drug^5^, is a much more potent inhibitor (K_I_ of 83 nM) of TweCAδ compared to **36** (Table 1).

(iv) The most effective TweCAδ inhibitors identified in this MTC/DTC panel were the MTC **12** (K_I_ of 67.7 nM) and the DTC **27** (K_I_ of 93.6 nM). These compounds incorporate scaffolds rather similar to those present in other investigated compounds, which were, however, much less effective as inhibitors of this enzyme. For example, **12** has two methoxy moieties on the scaffold of **11**, but there is a difference of activity of 14.7-fold between the two MTCs. The R-enantiomer **27** was on the other hand 5.9 times a more effective inhibitor compared to the S-enantiomer **28**. All these data show that small changes in the structure or the stereochemistry of a DTC/MTC lead too dramatic changes of affinity for the target enzyme.

(v) With a few exceptions, TweCAδ was less sensitive to this class of CAIs compared to the α-CAs hCA I and II (Table 1). There are several X-ray crystal structures that demonstrate that the DTCs (and presumably also the MTCs) bind to the metal ion in the CA active site by substituting the hydroxide nucleophile that is responsible for the catalytic activity of the enzyme^1^^,^^2^. Most probably, this is also the inhibition mechanism by which DTCs and MTCs interact with δ-CAs. However, this enzyme class is the least studied of the 7 CA genetic families, and there are no X-ray crystal structures or even homology models available for any δ-CAs.

**Figure 3.**
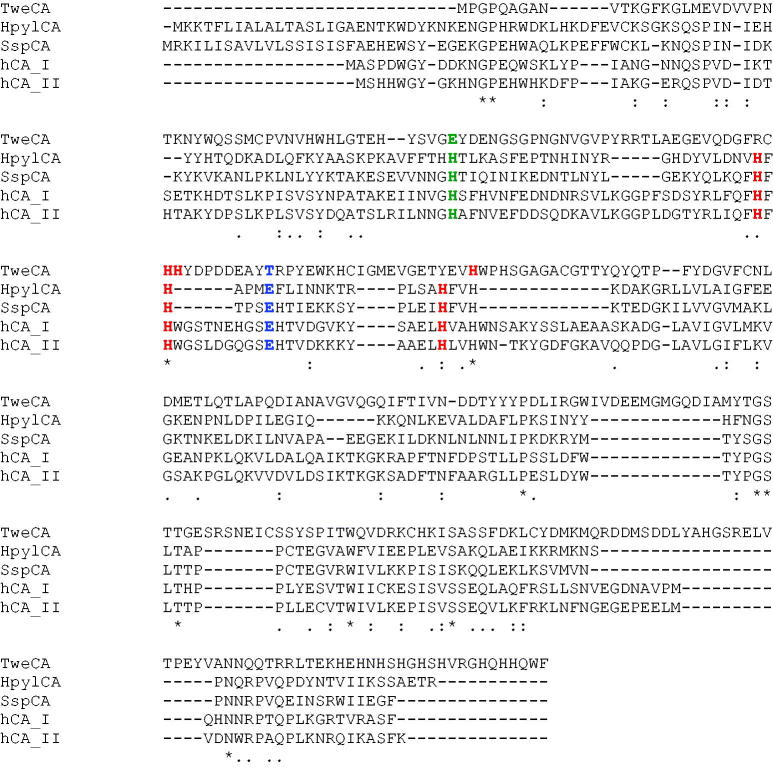
Multialignment of the TweCAδ amino acid sequence with those of bacterial (HpylCA, α-CA from *Helicobacter pylori*, SspCA, α-CA from *Sulfurihydrogenibium yellowstonensis*) and human (hCA I and II) α-class enzymes. The zinc ligands of the α-CAs and the putative zinc ligands of TweCAδ are evidenced in red, whereas amino acid residues involved in the catalytic inhibition/mechanism (e.g. His64 and Asp106, hCA I numbering) are shown in green and blue, respectively.

We try to rationalise the obtained inhibition data based on the amino acid sequence of TweCAδ, which has been aligned with that of α-CAs for which the X-ray crystal structure is known, of bacterial (HpylCA, α-CA from *Helicobacter pylori*, SspCA, α-CA from *Sulfurihydrogenibium yellowstonensis*) or human origin (hCA I and II) (Figure 3). Data of Figure 3 show that for the α-CAs, the zinc ligands are three His residues (His94, 96, and119, hCA I numbering system), which align well for the bacterial and human enzymes, whereas the putative zinc ligands of TweCAδ do not align at all with those of the α-class enzyme. The same is true for other amino acid residues from the α-CAs, such as the proton shuttle (His64) which is an Asp residue in TweCAδ, or residues 106 (a conserved Asp residue in all α-CAs), which is a Thr in TweCAδ. Based on these data it is obvious that it is not possible to rationalise the observed SAR with mono- and di-thiocarbamates based only on the sequence of the enzyme, without a homology model or better, an X-ray crystal structure of the diatom enzyme.

## Conclusions

The first inhibition study of a δ-CA with mono- and di-thiocarbamates, classes of CAIs recently discovered, was reported. TweCAδ from the marine diatom *T. weissflogii* was not particularly sensitive to inhibition by these classes of compounds. Many of the mono- and di-thiocarbamates did not show inhibitory action up to 20 µM, whereas some aliphatic, heterocyclic, and aromatic inhibited this enzyme in the low micromolar range. Several MTCs/DTCs incorporating the piperazine ring effectively inhibited TweCAδ with K_I_s in the range of 129–791 nM. The most effective inhibitors identified were 3,4-dimethoxyphenyl-ethyl-mono-thiocarbamate (K_I_ of 67.7 nM) and the *R*-enantiomer of the nipecotic acid DTC (K_I_ of 93.6 nM). Such inhibitors can now be used as molecular probes to investigate the role of this enzyme in the carbon fixation processes in diatom marine organisms that are responsible for removing large amounts of CO_2_ from the atmosphere.
